# Supplementary material for: Adaptation after vastus lateralis denervation in rats demonstrates neural regulation of joint stresses and strains
Source: eLife. 2018 Sep 3;7:e38215. doi: 10.7554/eLife.38215 (PMC6150696; doi:10.7554/eLife.38215)
Supplement: Transparent reporting form [file elife-38215-transrepform.docx]

***eLife’s* transparent reporting form**

We encourage authors to provide detailed information *within their submission* to facilitate the interpretation and replication of experiments. Authors can upload supporting documentation to indicate the use of appropriate reporting guidelines for health-related research (see [EQUATOR Network](http://www.equator-network.org/%20)), life science research (see the [BioSharing Information Resource](https://biosharing.org/)), or the [ARRIVE guidelines](http://www.plosbiology.org/article/info:doi/10.1371/journal.pbio.1000412) for reporting work involving animal research. Where applicable, authors should refer to any relevant reporting standards documents in this form.

If you have any questions, please consult our Journal Policies and/or contact us: [editorial@elifesciences.org](mailto:editorial@elifesciences.org).

**Sample-size estimation**

- You should state whether an appropriate sample size was computed when the study was being designed
- You should state the statistical method of sample size computation and any required assumptions
- If no explicit power analysis was used, you should describe how you decided what sample (replicate) size (number) to use

Sample sizes were determined empirically. The number of animals included in the analysis was limited by technical challenges, mainly due to precise surgical techniques and the longitudinal recordings required for this study; i.e. each animal was initially trained for treadmill locomotion, then implanted with EMG electrodes during surgery, after ~10 days of recovery baseline was recorded, then denervation was performed, and then data were recorded over a period of 7 weeks (~2.5 months for each animal, see Materials and Methods -> Experimental Protocol). For each session, we recorded two minutes of locomotor activity, allowing us to obtain a good number of strides and limit fatigue. The number of strides included in the analysis was limited by the inclusion criteria described in section Materials and Methods -> Data acquisition and processing.

Please outline where this information can be found within the submission (e.g., sections or figure legends), or explain why this information doesn’t apply to your submission:

**Replicates**

- You should report how often each experiment was performed
- You should include a definition of biological versus technical replication
- The data obtained should be provided and sufficient information should be provided to indicate the number of independent biological and/or technical replicates
- If you encountered any outliers, you should describe how these were handled
- Criteria for exclusion/inclusion of data should be clearly stated
- High-throughput sequence data should be uploaded before submission, with a private link for reviewers provided (these are available from both GEO and ArrayExpress)

Please outline where this information can be found within the submission (e.g., sections or figure legends), or explain why this information doesn’t apply to your submission:

N=6 animals included in the study. Ns=65 average number of strides for each recording session and animal (this number varies across animals and recording sessions based on the inclusion criteria described in Materials and Methods -> Data acquisition and processing).

Precise information about number of animals and number of strides is provided in the figure legend corresponding to each analysis. No outliers were excluded from the analysis.

**Statistical reporting**

- Statistical analysis methods should be described and justified
- Raw data should be presented in figures whenever informative to do so (typically when N per group is less than 10)
- For each experiment, you should identify the statistical tests used, exact values of N, definitions of center, methods of multiple test correction, and dispersion and precision measures (e.g., mean, median, SD, SEM, confidence intervals; and, for the major substantive results, a measure of effect size (e.g., Pearson's r, Cohen's d)
- Report exact p-values wherever possible alongside the summary statistics and 95% confidence intervals. These should be reported for all key questions and not only when the p-value is less than 0.05.

We analyzed longitudinal data (both EMG and kinematics) by means of Linear Mixed Effects Models and post-hoc tests with Bonferroni corrections. This allowed us to consider single-trial data (i.e. each locomotor stride) as opposed to group averages, hence exploiting the full power of our dataset. We analyzed muscle mass data by means of paired t-tests with Bonferroni correction. Detailed information on statistical tests can be found in Materials and methods -> Statistical analysis.

Please outline where this information can be found within the submission (e.g., sections or figure legends), or explain why this information doesn’t apply to your submission:

(For large datasets, or papers with a very large number of statistical tests, you may upload a single table file with tests, Ns, etc., with reference to sections in the manuscript.)

**Group allocation**

- Indicate how samples were allocated into experimental groups (in the case of clinical studies, please specify allocation to treatment method); if randomization was used, please also state if restricted randomization was applied
- Indicate if masking was used during group allocation, data collection and/or data analysis

This is a repeated measure design; i.e. each animal was recorded longitudinally before and at various time points after muscle denervation. Additional details on the study protocol can be found in Materials and methods -> Experimental protocol.

Please outline where this information can be found within the submission (e.g., sections or figure legends), or explain why this information doesn’t apply to your submission:

**Additional data files (“source data”)**

- We encourage you to upload relevant additional data files, such as numerical data that are represented as a graph in a figure, or as a summary table
- Where provided, these should be in the most useful format, and they can be uploaded as “Source data” files linked to a main figure or table
- Include model definition files including the full list of parameters used
- Include code used for data analysis (e.g., R, MatLab)
- Avoid stating that data files are “available upon request”

We have uploaded data files and data analysis code for Figures, 2,3,4,5,6 and Figure 3 supplemental figure 1, and Figure 6 supplemental figure 1.

Please indicate the figures or tables for which source data files have been provided:
